# Outcomes of the roll-in cohort of the Amulet IDE trial of left atrial appendage occlusion

**DOI:** 10.1016/j.hroo.2022.07.004

**Published:** 2022-07-20

**Authors:** Dhanunjaya Lakkireddy, David Thaler, Christopher R. Ellis, Vijendra Swarup, Lars Sondergaard, John Carroll, Michael R. Gold, James Hermiller, Hans-Christoph Diener, Boris Schmidt, Lee MacDonald, Moussa Mansour, Brijeshwar Maini, Jordan A. Anderson, Ryan Gage, Stephan Windecker, Amulet IDE Investigators

**Affiliations:** ∗ Kansas City Heart Rhythm Institute and Research Foundation, Overland Park, Kansas; † Tufts Medical Center, Boston, Massachusetts; ‡ Vanderbilt Heart Institute, Nashville, Tennessee; § Arizona Cardiovascular Research Center, Phoenix, Arizona; ¶ Righospitalet, Copenhagen, Denmark; ‖ University of CO Hospital, Anschultz Medical Campus, Aurora, Colorado; ∗∗ Medical University of South Carolina, Charleston, South Carolina; †† St. Vincent Medical Group, Inc., Indianapolis, Indiana; ‡‡ University Duisburg-Essen, Essen, Germany; §§ Cardioangiologisches Centrum Bethanien, Frankfurt, Germany; ¶¶ South Denver Cardiology Associates P.C, Littleton, Colorado; ‖‖ Massachusetts General Hospital, Boston, Massachusetts; ∗∗∗ Delray Medical Center, Delray, Florida; ††† Abbott Structural Heart, Plymouth, Minnesota; ‡‡‡ Inselspital, University of Bern, Bern, Switzerland

**Keywords:** Amulet, Atrial fibrillation, Cardioembolic event, Left atrial appendage, Stroke, Stroke prevention

## Abstract

**Background:**

Left atrial appendage (LAA) occlusion is an alternative therapy to oral anticoagulants to reduce stroke risk in patients with nonvalvular atrial fibrillation (NVAF). The Amulet IDE trial compared the Amplatzer™ Amulet™ occluder (Abbott) with the Watchman™ 2.5 device (Boston Scientific) for LAA occlusion in patients with NVAF.

**Objective:**

The purpose of this study was to describe outcomes of the Amulet IDE trial roll-in cohort.

**Methods:**

At US sites up to 3 patients per implanter could be implanted with the Amulet occluder in the roll-in phase. The primary Endpoints in the Amulet IDE trial included safety (composite of procedure-related complications, all-cause death, or major bleeding at 12 months), effectiveness (composite of ischemic stroke or systemic embolism at 18 months), and rate of LAA occlusion at 45 days.

**Results:**

A total of 201 roll-in patients were enrolled. Device success occurred in 99% of patients, and device closure (residual jet ≤5 mm) was observed in 98.9% of patients at 45 days. The safety endpoint rate was numerically higher (worse) in the roll-in cohort compared to the randomized Amulet occluder cohort (18.4% vs 14.5%). Six patients (3.1%) experienced an ischemic stroke and 0 patients with a systemic embolism within 18 months, which was similar to the primary effectiveness endpoint rate in the randomized Amulet occluder cohort (2.8%).

**Conclusions:**

Despite lack of experience of the operators with the Amulet occluder in the roll-in phase, device implant success was high, a high rate of device closure was achieved, and low stroke rates were observed in patients with NVAF.

**Key Findings**

▪ Despite the limited experience of the operators participating in the Amulet IDE roll-in phase, device implant success was high, a high rate of device closure was achieved, and low stroke rates were observed in a population with a high risk of stroke and bleeding.
▪ Procedure-related complications are expected to decrease with increased implanter experience with the Amulet occluder.
▪ The Amulet occluder offers the advantages of left atrial appendage closure with the option to discharge without the use of oral anticoagulants.

## Introduction

Atrial fibrillation (AF) is the most common cardiac rhythm disorder and is associated with a 3- to 5-fold increased risk for thromboembolic stroke.^1^ Oral anticoagulation (OAC) therapy with non–vitamin K antagonist (VKA) oral anticoagulants (NOACs) is the standard of care for the prevention of thromboembolic events in patients with AF. However, many patients are not candidates for long-term OAC therapy because of a high risk of major bleeding, recurrent bleeding events, poor adherence related to medication adverse effects, and drug interactions.^2^

Evidence supporting left atrial appendage occlusion (LAAO) for stroke prophylaxis in patients with nonvalvular atrial fibrillation (NVAF) was provided in the pivotal randomized controlled trials PROTECT-AF (WATCHMAN Left Atrial Appendage System for Embolic PROTECTion in Patients With Atrial Fibrillation) and PREVAIL (Prospective Randomized Evaluation of the WATCHMAN LAA Closure Device in Patients With Atrial Fibrillation [AF] Versus Long Term Warfarin Therapy) comparing the Watchman^TM^ left atrial appendage (LAA) closure device (Boston Scientific, Maple Grove, MN) to the VKA warfarin. Accordingly, LAAO is considered a viable alternate to medical therapy for thromboembolic stroke reduction in patients with AF who are poor candidates for long-term OAC.

The Amplatzer™ Amulet™ occluder (Abbott Medical, Plymouth, MN) received CE (Conformité Européene) Mark in 2013 and US FDA (Food and Drug Administration) approval in 2021. The Amulet IDE trial was a prospective, global, multicenter, randomized, controlled, noninferiority trial that directly compared the safety and effectiveness of the Amulet occluder to the FDA-approved and commercially available Watchman LAA closure 2.5 device.^3^^,^^4^ To provide Amulet occluder implant experience at US sites before to randomization, a roll-in phase was used. Here we present the safety and efficacy of the Amulet occluder in the roll-in patient cohort.

## Methods

The Amulet IDE trial (AMPLATZER™ Amulet™ Left Atrial Appendage Occluder Randomized Controlled Trial; ClinicalTrials.gov Identifier: NCT02879448) compares the safety and effectiveness of the Amulet occluder with the Watchman 2.5 device for thromboembolic stroke prevention in patients with NVAF. Details about the design of the Amulet IDE trial have been reported previously.^3^^,^^4^ The protocol was approved by the institutional review board at each participating center and adhered to the Helsinki guidelines. All patients provided written informed consent.

Intended users of the LAAO devices in the Amulet IDE trial included interventional cardiologists and electrophysiologists trained on both devices (Amulet^TM^ occluder and Watchman^TM^ device) and must have performed ≥25 interventional cardiac procedures that involved percutaneous puncture through an intact interatrial septum.^3^ For implanting facilities to participate, the following were required: hospital with an established structural heart disease and/or electrophysiology program; hospital with a cardiac catheterization laboratory or electrophysiology laboratory with fluoroscopy capability; imaging (ie, transesophageal echocardiography [TEE]) with echocardiography support; and anesthesiology support for administration of general anesthesia specific to this procedure (as necessary).

Because non-US sites in the trial had access to the Amulet occluder since 2013, only US sites were allowed to enroll roll-in patients, Before randomization at US sites, up to 3 patients per sponsor-approved implanter could be implanted with the Amulet occluder as part of the roll-in phase to gain hands-on-experience, before randomizing patients in the trial. Adverse events were adjudicated by an independent clinical events committee, and LAAO was assessed by an independent core laboratory based on the 45-day TEE.

### Patient selection

Roll-in patients met the same eligibility criteria, had the same data collection requirements, and underwent the same primary endpoint assessment as randomized patients. Key inclusion criteria included documented paroxysmal, persistent, or permanent NVAF; high risk of stroke or systemic embolism defined as CHADS_2_ score ≥2 or CHA_2_DS_2_-VASc score ≥ 3; and deemed by the investigator to be suitable for short-term VKA therapy but unable to receive long-term OAC.

### Amulet occluder procedure

The Amulet occluder was implanted as previously described.^3^^,^^4^ The procedure was guided by TEE and fluoroscopy. The protocol required that patients be discharged on dual antiplatelet therapy (DAPT) (aspirin and clopidogrel) or aspirin and OAC therapy at investigator discretion. If, at the 45-day visit, TEE showed adequate closure of the LAA (residual jet ≤5 mm) and absence of device-related thrombus (DRT), cessation of OAC was required and patients were instructed to take DAPT until the 6-month visit. Cessation of clopidogrel was required at the 6-month visit, and aspirin was to be continued indefinitely.

Follow-up clinical assessments occurred at discharge, and after 45 days, 3 months (phone), 6 months, 9 months (phone), 12 months, and 18 months. Patients will continue to be followed for 5 years. A Questionnaire to Verify Stroke Free Status (QVSFS) was administered at all study visits. All patients who provided positive answers underwent further evaluation, including neurological assessment and imaging, as necessary.

Device success was defined as device deployed and implanted in the correct position.^5^ Technical success was defined as exclusion of the LAA with residual jet ≤5 mm postimplant with no device-related complications.^5^ Procedural success was defined as technical success with no procedure-related complications.^5^

### Endpoints

The 3 primary endpoints defined in the Amulet IDE trial are (1) device closure, defined as residual jet around the device ≤5 mm at the 45-day TEE in which leak was graded as the single largest jet passed by the entirety of the dual mechanism device (mechanism of action); (2) a composite of procedure-related complications, all-cause death, or major bleeding (Bleeding Academic Research Consortium [BARC] types 3–5) through 12 months (safety); and (3) a composite of ischemic stroke or systemic embolism through 18 months (effectiveness). Procedure-related complications were defined as adverse events adjudicated as procedure related and required either surgical or percutaneous intervention. Major bleeding was defined as BARC type 3 or greater.^6^

Prespecified secondary endpoints included (1) a composite of all stroke (ischemic or hemorrhagic), systemic embolism, or cardiovascular/unexplained death at 18 months; and (2) major bleeding at 18 months.

### Statistical analysis

Baseline demographic and clinical characteristics are summarized using descriptive statistics. Categorical variables are summarized with percentages, and continuous variables are summarized as mean ± SD. The Kaplan-Meier method was used to calculate event rates for mortality, the composite of ischemic stroke, systemic embolism, and cardiovascular death, and DRT.

## Results

**Figure 1:**
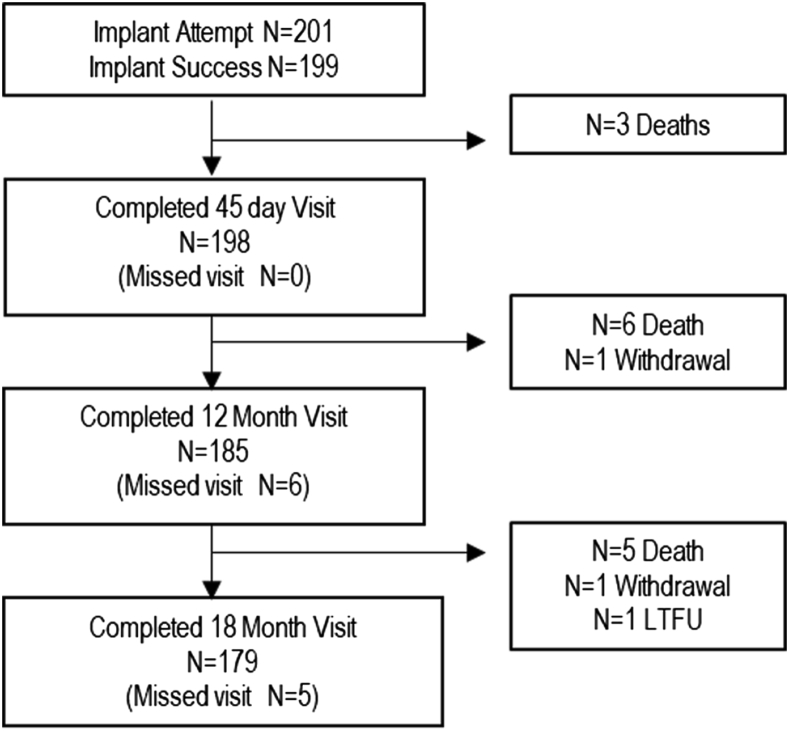
Flowchart of patient follow-up. The number of patients who completed each follow-up visit in the roll-in cohort. Through the 18-month follow-up, visit compliance (actual follow-up rate) was 95.7%. Fourteen patients were lost due to death, 2 withdrew, and 1 was lost to follow-up (LTFU).

A total of 201 roll-in patients were enrolled between August 2016 and November 2018. Implantation procedures were performed by 133 investigators at 82 US sites. Implanters had performed an average of 82 Watchman^TM^ device cases before the Amulet IDE trial, with 58 of the 133 investigators having no previous experience with either the Amplatzer^TM^ Cardiac Plug (first-generation Amplatzer LAAO device) or Amulet^TM^ occluder. Also, sites had an average of 8 Watchman device cases per month and 162 annual number of patients screened for LAAO before the Amulet IDE trial. Figure 1 details patient follow-up. Through the 18-month follow-up, visit compliance (actual follow-up rate) was 95.7%; 14 patients were lost due to death, 2 withdrew, and 1 was lost to follow-up.

### Patient characteristics

Patient demographics and clinical characteristics of the roll-in cohort are given in Table 1. The roll-in cohort had similar baseline characteristics as that of the randomized Amulet occluder cohort in the Amulet IDE trial.^4^ The roll-in cohort was 61.7% male (mean age 74.2 ± 7.5 years). Patients were at high risk for thromboembolic events, with mean CHA_2_DS_2_-VASc score of 4.6 ± 1.4, 10.9% prevalence of previous transient ischemic attack (TIA), and 20.9% prevalence of previous stroke. Most patients were at high risk for major bleeding events, with a HAS-BLED score of 3.3±1.0, and previous major and/or minor bleeding was reported in 75.1% of patients.

**Table 1:** Patient demographics and clinical history of the roll-in cohort (N = 201)

Age (y)	74.2 ± 7.5
Male	61.7 (124)
Weight (kg)	90.1 ± 21.6
AF type	
Paroxysmal	54.2 (109)
Persistent	29.9 (60)
Permanent	15.9 (32)
CHA_2_DS_2_-VASc score	4.6 ± 1.4
HAS-BLED score	3.3 ± 1.0
Previous stroke	20.9 (42)
Previous transient ischemic attack	10.9 (22)
Previous bleeding	75.1 (151)
Previous myocardial Infarction	15.9 (32)
Warfarin use	
≥1 y	43.3 (87)
<1 y	17.9 (36)
None	38.8 (78)
NOAC use	
≥1 y	30.3 (61)
<1 y	32.3 (65)
None	37.3 (75)

Values are given as mean ± SD or % (n).

AF = atrial fibrillation; NOAC = non–vitamin K antagonist oral anticoagulant.

### Procedural outcomes

Procedural outcomes are given in Table 2. Most procedures (99% [n = 199]) were performed under general anesthesia, and 2 implants were performed under conscious sedation as a result of physician preference. Total procedural time, defined as the time elapsed from vascular access with the delivery system to the time the delivery system was removed, was 46.4 ± 23.6 minutes compared to 39.9 ± 23.8 minutes in the randomized Amulet occluder cohort (absolute difference 6.5 minutes). Although this cohort represents the early Amulet occluder experience in the United States, most of cases (86.1%) were successful with the use of 1 device. Device success was 99.0% (199/201) compared to 98.4% in the randomized Amulet occluder cohort (absolute difference 0.6%). Two patients did not receive an Amulet occluder due to LAA anatomic issues. Of these patients, 1 patient did not receive any LAAO device after the Amulet occluder implant attempt was unsuccessful due to an appendage that was too small. The second patient received a Watchman device at the physician’s discretion after the Amulet occluder implant attempt was unsuccessful. Technical success was 97.5% (196/201), and procedural success was 95.5% (192/201), similar to the randomized Amulet occluder cohort results (97.2% and 96.0%, respectively).

**Table 2:** Procedural outcomes

Cohort	Roll-in (N = 201)	Randomized (N = 915)	Absolute difference
Procedural duration (min)	46.4 ± 23.6	39.9 ± 23.8	6.5 min
Fluoroscopy time (min)	15.7 ± 7.8	13.8 ± 8.9	1.9 min
General anesthesia	99.0 (199)	92.1 (843)	6.9%
No. of devices attempted	1.2 ± 0.5∗	1.2 ± 0.5	0.0 devices
0	0.0 (0)	0.4 (4)	0.4
1	86.1 (173)	81.6 (747)	4.5
2	10.9 (22)	14.9 (136)	4.0
3+	3.0 (6)	3.1 (28)	0.1%
Device success	99.0 (199)	98.4 (900)	0.6%
Technical success	97.5 (196)	97.2 (889)	0.3%
Procedural success	95.5 (192)	96.0 (878)	0.5%
Site reported residual jet >5 mm postimplant	0.0 (0)	0.0 (0)	0.0%
Device size implanted (mm)	Roll-in (N = 199)	Randomized (N = 900)	Absolute difference
16	1.0 (2)	0.7 (6)	0.3%
18	7.5 (15)	3.7 (33)	3.8%
20	8.0 (16)	8.0 (72)	0.0%
22	17.6 (35)	23.9 (215)	6.3%
25	30.2 (60)	36.6 (329)	6.4%
28	19.6 (39)	18.8 (169)	0.8%
31	9.0 (18)	6.3 (57)	2.7%
34	7.0 (14)	2.1 (19)	4.9%

Values given as mean ± SD or % (n) unless otherwise indicated. Absolute differences are provided from the roll-in cohort vs the results presented in the randomized Amulet^TM^ occluder cohort.^4^

Procedural duration was defined as the time elapsed from vascular access with the delivery system to the time the delivery system was removed. Device success is defined as device deployed and implanted in correct position. Technical success is exclusion of the left atrial appendage with site reported residual jet ≤5 mm and no device-related complications through discharge or 7 days, whichever is earlier. Procedural success is technical success with no procedure-related complications.

∗ An adjacent (smaller or larger) or same size of device was successfully implanted after the previous device attempt.

### Antithrombotic medications

**Figure 2:**
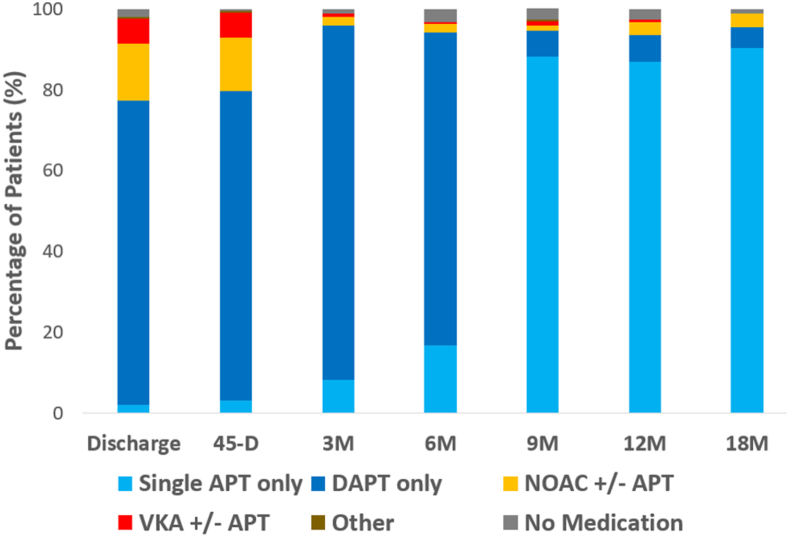
Antithrombotic medication usage through 18 months in the roll-in cohort. Data are given as count (%) of all patients (per time point). No patients were mandated to discharge on oral anticoagulant (OAC) due to the protocol. At discharge, 75.4% of patients were on dual antiplatelet therapy (DAPT) (20.1% were on OAC at the physician’s discretion). Most continued taking DAPT until the 6-month follow-up visit. At the 9-, 12-, and 18-month follow-up visits, ∼90% of patients were on single antiplatelet therapy. APT = antiplatelet therapy; NOAC = non–vitamin K antagonist oral anticoagulant; VKA = vitamin K antagonist.

The percentage of patients on various antithrombotic medical therapy regimens is shown in Figure 2. Similar to the randomized Amulet occluder cohort, the majority (75.4%) of roll-in patients were discharged on DAPT (roll-in: 75.4% vs randomized: 75.7%^4^), and 20.1% were discharged on OAC at the physician’s discretion (not protocol mandated). Most continued taking DAPT until the 6-month follow-up visit. At the 9-, 12-, and 18-month follow-up visits, ∼90% of roll-in patients were on single antiplatelet therapy.

### Primary mechanism of action endpoint

As shown in Figure 1, 198 patients completed the 45-day visit. Of these patients, 1 did not have a device implanted, 1 was implanted with a Watchman device, and 2 TEEs were unable to be uploaded to the echocardiography core laboratory. Therefore, 194 TEEs were reviewed by the core laboratory, and 180 were suitable for primary endpoint analysis. Device closure was observed in 98.9% of patients (Table 3). No flow around the device (complete occlusion) was observed in 61.7% of patients, flow ≤5 mm was observed in 37.2% of patients, and 2 patients (1.1%) had flow >5 mm. These results are similar to those observed in the randomized Amulet occluder cohort, with absolute differences of 1.3%, 1.4%, and 0.0% for flow of 0, ≤5 mm, and >5 mm, respectively.

**Table 3:** Primary and secondary endpoints

Cohort	Roll-In	Randomized	Absolute difference (%)
Primary mechanism of action endpoint: Peridevice leak at 45 d	98.9 (178/180)	98.9 (792/801)	0.0
None	61.7 (111/180)	63.0 (505/801)	1.3
≤5 mm	37.2 (67/180)	35.8 (287/801)	1.4
>5 mm	1.1 (2/180)	1.1 (9/801)	0.0
Primary safety endpoint at 1 y	18.4 (37/201)	14.5 (131/903)	3.9
Procedure-related complications	3.5 (7/201)∗	4.5 (41/903)	1.0
All-cause death	4.5 (9/201)	3.9 (35/903)	0.6
Major bleeding (BARC type 3 or greater)	15.6 (31/201)	10.6 (95/903)	5.0
Nonprocedure-related major bleeding	12.2 (24/201)	7.9 (70/903)	4.3
Primary effectiveness endpoint at 18 mo	3.1 (6/201)	2.8 (25/934)	0.3
Ischemic stroke	3.1 (6/201)	2.5 (22/934)	0.6
Systemic embolism	0.0 (0/201)	0.3 (3/934)	0.3
Major bleeding at 18 mo	16.1 (32/201)	11.6 (105/917)	4.5
Stroke, systemic embolism, cardiovascular/unexplained death at 18 mo	7.0 (14/201)	5.6 (50/915)	1.4
All stroke	3.6 (7/201)	2.7 (24/915)	0.9
Systemic embolism	0.0 (0/201)	0.3 (3/915)	0.3
Cardiovascular/unexplained death	4.0 (8/201)	3.1 (28/915)	0.9

Data are given as Kaplan-Meier estimated rate (n/N) or % (n/N) unless otherwise indicated. Absolute differences are provided from the roll-in cohort vs the results presented in the randomized Amulet^TM^ occluder cohort^4^.

BARC = Bleeding Academic Research Consortium.

∗ Pericardial effusion/tamponade (n = 3), device embolization (n = 1), major bleeding (n = 1), intracerebral hemorrhage (n = 1), and vascular access site bleeding (n = 1).

### Primary safety endpoint

A total of 37 patients met ≥1 components of the primary safety endpoint through 12 months (Kaplan-Meier composite estimate 18.4%) (Table 3). This was higher than what was reported in the randomized Amulet occluder cohort (14.5%). The Kaplan-Meier estimates for procedure-related complications, all-cause death, and major bleeding were 3.5% (n = 7 patients), 4.5% (n = 9 patients), and 15.6% (n = 31 patients), respectively, in the roll-in cohort.

Seven patients experienced procedure-related complications, including pericardial effusion (n = 3), device embolization (n = 1), major bleeding (n = 1), intracerebral hemorrhage from an injury to the head with unknown relation to the implant procedure (n = 1), and vascular access site bleeding (n = 1). The pericardial effusion events all occurred within 2 days of the procedure (before hospital discharge). All 3 patients had only 1 device attempt. Two patients were discharged on OAC and 1 patient was discharged on DAPT. One patient experienced chest discomfort and shortness of breath postprocedure, and 2 patients had no symptoms. All 3 pericardial effusions resolved without sequalae: 2 of the events drained percutaneously without the need for surgery, 1 patient underwent pericardial window, and none required open cardiac surgery. The device embolization event occurred 1 day postprocedure (before hospital discharge) when the radiograph showed the device in the abdominal aorta. The patient experienced no symptoms, and the device was successfully retrieved and resolved without sequalae.

The primary safety endpoint composite was primarily driven by the major bleeding events, which was also the largest absolute difference from the randomized Amulet occluder cohort (5.0%). Of the 31 major bleeding events in the roll-in cohort, 7 occurred ≤7 days after the procedure, 7 between 8 and 30 days postprocedure, and an additional 17 events occurred 30 days postprocedure. Most (78%) major bleeding events were adjudicated as unrelated to the procedure or device.

There were 9 deaths (4.5%) within 12 months in the roll-in cohort compared to 35 deaths (3.9%) in the randomized Amulet occluder cohort. Five deaths were adjudicated as cardiovascular in cause, and 4 were adjudicated as noncardiovascular.

### Primary effectiveness endpoint

A total of 6 patients experienced an ischemic stroke and 0 patients experienced a systemic embolism within 18 months (Kaplan-Meier estimate 3.1%), corresponding to an ischemic stroke annualized rate of 2.1% per year. These results (Table 3) are similar to those of the randomized Amulet occluder cohort (2.8% Kaplan-Meier estimate and 1.7% per year annualized rate). One ischemic stroke occurred 4 days postprocedure and was adjudicated as device and procedure related. Five additional strokes occurred in 5 patients within 18 months (0, 57, 183, 266, and 276 days postprocedure), and all were adjudicated as not procedure related with an unknown relationship to the device. At the time of ischemic stroke, 3 patients were taking aspirin only, and 3 patients were taking DAPT. There were no DRTs, and flow ≤5 mm was observed in all these patients. No patients died from ischemic stroke, and no systemic embolisms were reported.

### Secondary endpoints

Kaplan-Meier estimates of the rate of major bleeding, defined as BARC type 3 or greater, at 18 months are given in Table 3. Thirty-two patients experienced ≥1 major bleeding events at 18 months, resulting in an 18-month estimated event rate of 16.1%, which is higher than the rate in the randomized Amulet occluder cohort of 11.6% (absolute difference 4.5%). The rates of stroke, systemic embolism, and cardiovascular/unexplained death at 18 months are given in Table 3. Six patients experienced an ischemic stroke, and 1 patient experienced a hemorrhagic stroke. No patient experienced systemic embolism. The event rate for this endpoint was 7.0%.

### Descriptive endpoints

The descriptive endpoints are given in Table 4. Eight patients had a DRT (4.0%). All events were identified as part of regular follow-up (6 at the 45-day visit, 1 at the 6-month visit [did not have a DRT present at 45-day TEE]), and 1 at the 12-month visit [unevaluable 45-day TEE due to poor image quality]). All patients were taking antiplatelet medication at the time the DRT was identified (7 DAPT, 1 aspirin only). None of the patients with DRT experienced an ischemic stroke within 18 months.

**Table 4:** Descriptive endpoints at 18 months

Cohort	Roll-in (N = 201)	Randomized (N = 915)	Absolute difference (%)
Device-related thrombus	4.0 (8)	3.3 (30)	0.7
Transient ischemic attack	2.5 (5)	1.5 (15)	1.0
Hemorrhagic stroke	0.5 (1)	0.3 (3)	0.2

Data are given as % (n) unless otherwise indicated. Absolute differences are provided from the roll-in cohort vs the results presented in the randomized Amulet^TM^ occluder cohort.^4^

Five TIAs occurred in 5 patients, for an annualized rate of 1.7% per year. One patient experienced a hemorrhagic stroke, for an annualized rate of 0.3% per year. DRT, TIA, and hemorrhagic stroke occurred at similar rates in the randomized Amulet occluder cohort (3.3% DRT, 1.5% TIA, 0.3% hemorrhagic stroke).

## Discussion

The learning curve effects during the initial phases of cardiovascular device adoption are well documented. Studies have shown there is an initial rapid learning phase for implant success, with improvement observed over time.^7^, ^8^, ^9^ Although roll-in results have not been reported for the Watchman^TM^ device, the PROTECT-AF trial mostly had new operators implanting the Watchman device. During early follow-up in PROTECT-AF, there were more primary safety periprocedural events in the Watchman device group compared to the warfarin control group (5.5% per year vs 3.6% per year).^10^ Also, Cruz-Gonzalez et al^11^ revealed increased periprocedural complications during initial experience with the first-generation Amplatzer^TM^ Cardiac Plug, which significantly decreased in frequency with an increase in operator experience. Although most implanters in the Amulet IDE had previous Watchman device experience (>50 cases), almost half of implanters in the roll-in portion of the Amulet IDE trial had no previous experience with either the Amplatzer Cardiac Plug or Amulet^TM^ occluder.

The Amulet occluder is approved for use in the United States. The dual-component design (lobe and disc) of the Amulet occluder may be more complex and contribute to a learning curve for Watchman device implanters, but it offers potential advantages for better sealing and the ability to seal complex LAA anatomies. The distal lobe offers the flexibility of placing the device in LAAs that are relatively shallow, LAAs with difficult angulations, or LAAs with proximal lobes, whereas the proximal disc provides a separate and second mechanism to enhance the likelihood of complete occlusion.

The results of this study showed that the initial experience during the roll-in phase of the Amulet IDE trial is comparable with regard to device effectiveness to the randomized Amulet occluder primary results reported,^4^ but safety and adverse events were higher in the roll-in phase. Many of the implanters were naïve to the Amulet occluder implantation procedure, although they may have had experience with other LAAO devices. Major findings of this early implant experience are (1) a high rate of device success; (2) a high degree of closure at 45 days; (3) higher (worse) composite safety endpoint rate compared to the randomized Amulet occluder cohort primarily driven by increased number of bleeding events; and (4) similar ischemic stroke/systemic embolism and DRT rates as observed in the randomized primary results.

The device success of the Amulet occluder was high (99%) despite the limited experience of the operators using this device and indicates that a prolonged learning curve is not needed in order to be proficient with implantation. The roll-in results further confirmed the high degree of closure at 45 days (98.9%), indicating the importance of a dual-seal mechanism in the Amulet occluder. The immediate and sustained closure allowed these patients to be treated without the need for OAC postprocedure at the physician’s discretion.

There was a higher (worse) composite safety endpoint rate in the roll-in results compared to the randomized results (roll-in: 18.4%; randomized Amulet occluder cohort: 14.5%). This was primarily driven by the increased number of bleeding events compared to what was observed in the randomized primary results portion of the trial (roll-in: 15.6% at 1 year and 16.1% at 18 months; randomized Amulet occluder cohort:10.6% at 1 year and 11.6% at 18 months). The bleeding outcomes were consistent with a patient population at high risk for bleeding (average HAS-BLED 3.3), and only a minority were related to the procedure or device at 1 year (3.4%). The decreased bleeding rate observed in the randomized portion of the trial suggests increased implanter experience may result in fewer bleeding events. Also, early implanter experience may have played a role in pericardial effusion events that occurred in the roll-in cohort, which was also observed in early operator experience during the randomized portion of the trial. Although the roll-in phase may illustrate a learning curve related to safety for new implanters, the device achieved similar results for effectiveness (ischemic stroke/systemic embolism composite) between the roll-in cohort and randomized Amulet occluder cohort (roll-in: 3.1%; randomized Amulet occluder cohort: 2.8%) and DRTs (roll-in: 4.0%; randomized Amulet occluder cohort: 3.3%).

This early experience of novice operators is encouraging. It demonstrates new operators can implant the recently approved Amulet occluder with a high degree of success and device closure while also achieving low stroke rates in patients with NVAF. As demonstrated in the randomized Amulet occluder cohort, procedure-related complications are expected to decrease with increased implanter experience with the Amulet^TM^ occluder. Finally, the postprocedural medical management with DAPT, instead of OAC, may offer a safety advantage.

### Study limitations

This early experience consists of a relatively small cohort of patients. Even though many of these investigators were Amulet occluder naïve, most had experience in transseptal punctures, LA navigation, and LAA closure with the Watchman device.

## Conclusion

The roll-in experience of the Amulet IDE trial showed the Amulet^TM^ LAA occluder safely and effectively achieves high occlusion without the use of OAC medication at discharge. Increased implanter experience with the Amulet occluder is expected to decrease procedure-related complications.

## Acknowledgments

The authors would like to thank all investigators and institutions participating in the roll-in portion of the Amulet IDE trial, and Hong Zhao, PhD, and Deepika Morishetti, MS (Abbott), for their contributions to data analysis.
